# Mobile applications for pain management: an app analysis for clinical usage

**DOI:** 10.1186/s12911-019-0827-7

**Published:** 2019-05-30

**Authors:** Peng Zhao, Illhoi Yoo, Robert Lancey, Ebby Varghese

**Affiliations:** 1 Informatics Institute, University of Missouri, Columbia, MO USA; 2 Department of Health Management and Informatics, School of Medicine, University of Missouri, Five Hospital Dr., CE718 Clinical Support and Education Building (DC006.00), Columbia, MO 65212 USA; 3 Division of General Internal Medicine, Department of Medicine, School of Medicine, University of Missouri, Columbia, MO USA; 4 Department of Physical Medicine and Rehabilitation, School of Medicine, University of Missouri, Columbia, MO USA

**Keywords:** Pain management, Chronic pain, Mobile applications, Mobile devices

## Abstract

**Background:**

Pain is the most common and distressing symptom for patients in all clinical settings. The dearth of health informatics tools to support acute and chronic pain management may be contributing to the chronic pain and opioid abuse crises. The purpose of this study is to qualitatively evaluate the content and functionality of mobile pain management apps.

**Methods:**

The Apple App Store and the Google Play Store were searched to identify pain management apps. The inclusion criteria were as follows: (1) that apps include a pain diary function allowing users to record pain episodes, (2) are available in either Apple App Store or Google Play Store, and (3) are available in the English language. We excluded apps if they were limited to only specific forms of pain or specific diseases.

**Results:**

A total of 36 apps met the inclusion criteria. Most of the apps served as pain diary tools to record the key characteristics of pain. The pain diary features of the apps were grouped into nine categories: the recordings of pain intensity, pain location, pain quality, pain’s impacts on daily life, other features of pain, other related symptoms, medication, patients’ habits and basic information, and other miscellaneous functions. The apps displayed various problems in use. The problem of not involving healthcare professionals in app development has not been resolved. Approximately 31% of apps including a pain diary function engaged clinicians in app development. Only 19% involved end-users in development and then only in an ad-hoc way. Only one third of the apps supported the cross-platforms, none of the apps supported clinician access to graphical pain data visualization, none secured HIPAA compliance, and none endorsed the PEG tool for primary care physicians’ chronic pain management.

**Conclusions:**

Most of the 36 pain management apps demonstrated various problems including user interface and security. Many apps lacked clinician and end-user involvement in app development impacting the clinical utility of these apps. We could not find any pain apps suitable for clinical usage despite high demand from clinicians due to the US opioid crisis.

**Electronic supplementary material:**

The online version of this article (10.1186/s12911-019-0827-7) contains supplementary material, which is available to authorized users.

## Background

Pain is the most common and distressing symptom for patients in all clinical settings [1–3]. Johannes and colleagues estimated that 30% of adults in the United States (over 93 million people) suffer from chronic pain [4]. As a result, pain has had a profound economic impact due to direct health care costs and loss of productivity. Gaskin and Richard estimated that the total financial cost of pain to society in the United States ranges from $560 to $635 billion in 2010 dollars [5]. In addition, they found that the total cost of pain is much higher than the total costs of heart disease ($309 B), cancer ($243 B), and diabetes ($188 B) [5]. In fact, pain has been improperly managed in many cases. Opioids have been widely prescribed for pain [6] despite a lack of evidence for benefit in many conditions. In 2012 alone, 259 million opioid prescriptions were written in the United States [7]. As a result, over-prescription of opiates has contributed to the opiate abuse epidemic. According to the US Centers for Disease Control and Prevention (CDC), more than 22,000 people died of prescription opioids abuse alone in 2015 [8]. Rudd and colleagues reported a continuous increase in deaths from prescription opioid abuse and overdose in the United States for the years 2000–2015, and a recent sharp increase in illicit opioid overdose driven mainly by heroin and synthetic fentanyl [9]. More importantly, these two increases are interconnected and drive the opioid abuse crisis in the US [9].

Many studies have found that pain is commonly overlooked, and thus is undertreated and sometimes not treated at all [2, 3, 10, 11]. We believe that the lack of health informatics tools, especially features for acute and chronic pain management in electronic medical record (EMR) systems, could be a contributor to the chronic pain and opioid abuse crises because providers do not possess the information necessary to make data-driven medical decisions for pain management. The result of a lack of longitudinal pain data often leads physicians to under-prescribe and/or over-prescribe pain medicine such as opioids leading to improper pain management and even dependence, side-effects, and abuse/misuse of analgesic medications. One primary reason that EMR systems lack pain management tools is that pain data are obtained too sporadically to be useful for pain management. As a result, the development of mobile applications (apps) to monitor patients’ pain are badly needed. There are several main reasons for these needs.

First, to the best of our knowledge, all major EMR systems used in the USA do not support self-reported pain management data from patients. The absence of these features in EMR systems is problematic for optimal pain management. Self-reporting is imperative for pain management because patients uniquely experience pain and its effects during daily life and activities. Through self-reporting of pain information, patients may communicate their pain control data to their providers. Without self-report from patients, nurses and physicians cannot follow the patient’s pain levels throughout their entire inter-visit course and are forced to reply on asking “How is your pain?” every 3–6 months for chronic pain and 1–2 time(s) per day (inpatients) for acute pain, which does not convey the true picture of a patient’s pain course. Information gathered during patient self-reporting between clinic visits allows the provider to determine not only what medications would be appropriate but what symptoms may be amenable to interventional or physical therapy options. The power of patient self-reporting over real time using specific descriptors can drive evidence-based medicine in this field. Second, EMR systems do not include features supporting chronic pain management. In order to assess and manage chronic pain, physicians need to know the location of the pain, intensity, quality, and its impact on a patient’s activity and life in a structured format [12, 13]; the impact of this information is that the provider can discern whether the pain is episodic and or continuous. The current EMR systems, however, are not able to systematically record and retrieve this information thus making it more difficult for physicians to manage chronic pain. In short, all pain patients and clinicians suffer from these system problems.

There are a growing number of mobile pain management apps available in app stores such as the Apple App Store [14] and the Google Play Store [15]. At least 11 review articles have been published evaluating pain management apps since 2011 [16–26]. Among them, 3 studies reviewed apps for specific forms of pain such as low back pain [26], postoperative pain [27], and pediatric pain [23]. Despite many published app reviews for the past several years, there are four reasons why we conducted this study. First, the field of mHealth including pain management apps has rapidly changed so that apps are frequently updated with new features and new apps are released. For example, while a study published in 2011 (conducted in the summer in 2010) reported that 79% of the pain management apps were available for Apple iOS and 16% were for Google Android [16], a study published in 2015 (conducted in 2014) reported that 64.2% of the pain management apps were available for Google Android and only 9% were for Apple iOS [24], and a study published in 2017 showed that 90.6% of this type of apps were available for Android and 9.1% were for iOS [27].

Second, each of the 11 review studies found that although there were many pain management apps available to download in the app stores, virtually none of the apps fully satisfy clinicians’ expectations in terms of medical evidence-based content, grounding in clinical guideline for pain, or involvement of clinicians and chronic pain patients (as end users) in app development. Because these apps could play an important role in pain management, we set out to study whether current mobile pain management apps fulfill expectations for more effective chronic pain management.

Third, many of the 11 previous studies did not actually download and install apps for their studies. Rather, they evaluated apps based on only app descriptions. In our study, we purchased (as applicable), downloaded, and installed apps for this review on our mobile devices.

Last, we sought a pain management app for clinical usage with a set of clear requirements; (1) the app must support both of the two major mobile operating systems Apple iOS and Google Android, (2) clinicians must be able to access pain data for patient care in a clinic using either an app or the web showing pain data in a graphical format, (3) the app must be Health Insurance Portability and Accountability Act (HIPAA)-compliant in the USA (or similar one in other countries) ensuring the privacy and security of protected health information, and (4) the app must support the Pain, Enjoyment, and General Activity (PEG) scale [13] for assessing chronic pain. The PEG was mainly developed for primary care physicians unlike other scales such as the Chronic Pain Grade questionnaire (CPG) [28] and Brief Pain Inventory (BPI) [29, 30]. The PEG is derived from the Brief Pain Inventory (BPI), and is regarded as an “ultra-brief” (three-item) scale [13]. The three items (questions) of the PEG for chronic pain assessment are average pain level, interference with enjoyment of life, and interference with general activity. The rationale behind the adoption of the PEG instead of the CPG or BPI is that the PEG is a simple scale yet a statistically reliable and valid measure of chronic pain [6, 13, 31–33]; other chronic pain measures (even the BPI) consist of multiple-pages of pain-related questions, that are impractical for everyday chronic pain monitoring. To the best of our knowledge, no study has attempted to identify apps meeting these requirements even though there is high demand by clinicians for such an app. As a result, the aim of this study is to provide a descriptive evaluation of the clinical features available in pain management apps with a pain diary function and their various characteristics, and to identify apps that meet the requirements for clinical usage. The identification of those apps is the primary reason for this study because the value of a pain management app would be maximized if health care providers could use it for clinical purposes. However, if patients with chronic pain and clinicians were to use a pain management app that is not designed for clinical purposes, such an app may do more harm than good. This study will provide insights into future directions and opportunities in the development of mobile solutions to support pain management.

## Methods

### Data source and search strategy

In this study, we performed a review of mobile applications (apps) for pain management. A search with keyword “pain” was performed on February 20th, 2018 to identify pain management apps for Apple iOS and Google Android mobile operating systems in the Apple App Store [14] (via Vionza.com [34]) and the Google Play Store [15], respectively.

### Test devices

The Android test devices included a Samsung Galaxy Note 3 running Android 5.0 and a Samsung Galaxy Tab S2 tablet running Android 7.0. The iOS test devices were an iPhone 6 s, iPhone X, and iPad Pro running iOS 11.2.6, which was the latest version during the search.

### App inclusion and exclusion criteria

In this study, we included apps focusing on pain management. For inclusion in the review, apps had to:

- Include a pain diary function (allowing users to record pain episodes)
- Availability in either Apple App Store [14] or Google Play Store [15]
- English language compatibility

We excluded apps if they were limited to only specific forms of pain or specific diseases such as back pain and headache.

### Data extraction and abstraction

PZ (a trained research assistant) searched for apps, installed apps, and extracted app features. PZ and IY crosschecked features. When we disagreed on app features, we discussed until we reached a consensus. We spent several hours testing each of most apps but sometimes up to a week to determine how daily pain-related data are graphically represented on screen. We abstracted metadata from all included mobile apps into a Microsoft Excel spreadsheet. The name, platform, developer, developer’s country of residence, and price were extracted from app descriptions. Features evaluated in this study include pain diary items (such as pain intensity, pain location, pain quality, and its impacts on life and activities), other features of pain, medication, patients’ habits and basic health information, miscellaneous features, data visualization, healthcare professionals’ and patients’ involvement, and fulfillment of the four requirements for clinical usage. The extracted features were initially put into a Microsoft Excel spreadsheet in a dense format, and then converted into a matrix format for app analysis.

### Documentation of app problems

In addition to recording app features in an Excel spreadsheet, PZ documented all problems and issues in the apps, and IY confirmed them. We determined that an app had a problem if we had significant difficulties using the features of the app. These difficulties include repeated app crashes, unavailable app servers (e.g., required registration was impossible), nonfunctional buttons, navigation problems (e.g., hard to go back the home screen), substandard user interface (too small font size, too many colors or patterns, and/or too many functions on a small screen), etc.

## Results

### Application selection

**Fig. 1:**
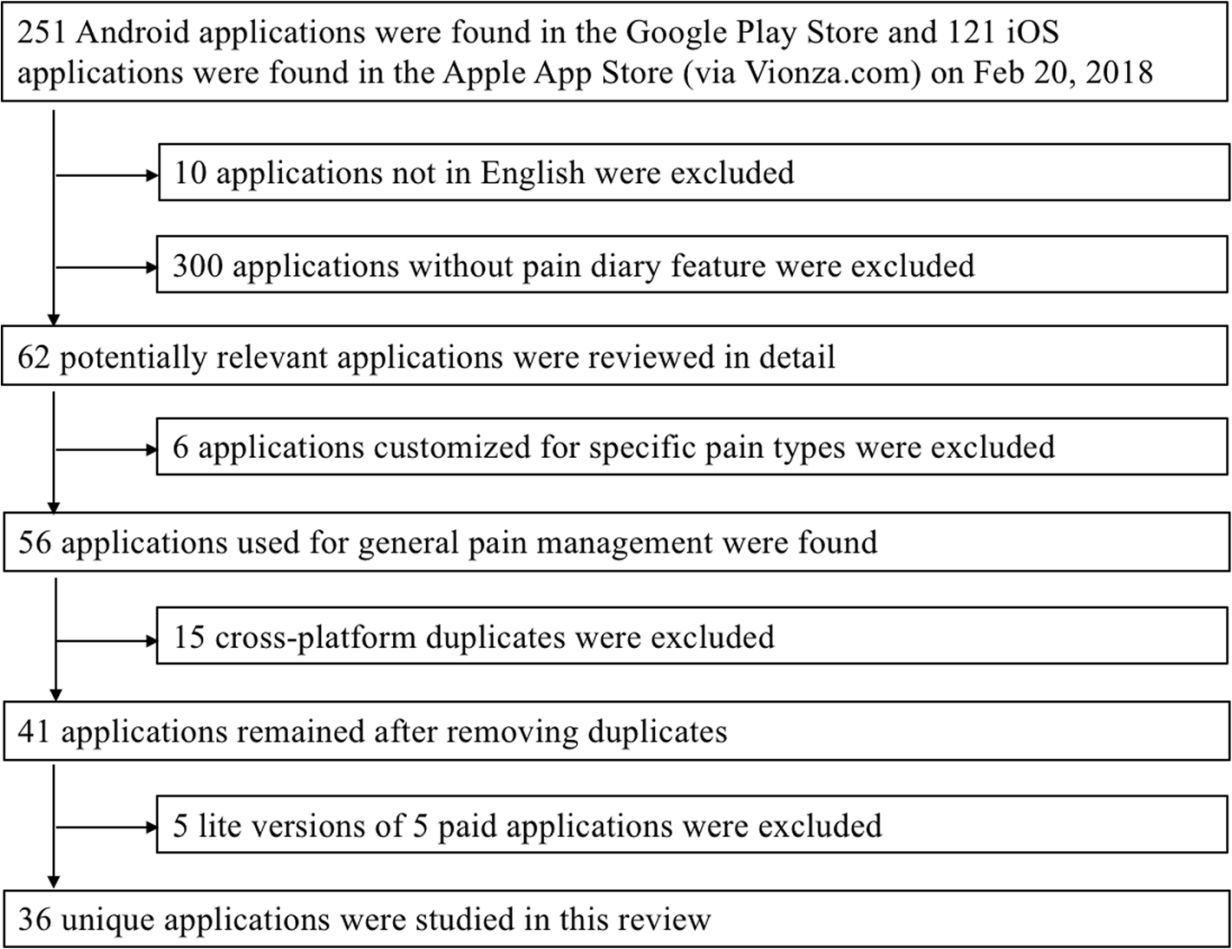
Trial flow diagram of identifying eligible apps

Figure 1 shows the process of identifying eligible apps. The query returned 251 and 121 apps, respectively. After removing 10 non-English apps, the remaining 362 apps were reviewed based on their descriptions. Three hundred apps were removed because they did not include a pain diary. The remaining 62 apps were then installed on Android and iOS devices and were studied in detail. Six apps were filtered out because they only targeted specific pain types, including headache, migraine, and back pain. Fifteen apps were available on both iOS and Android. If an app is available for the iOS and Android, and the iOS and Android versions of the app contained the same functions, we treated the two versions as one single cross-platform app to reduce redundancy. Five free apps were lite versions of five paid apps. They were also treated as one single app to reduce redundancy. The resulting 36 unique apps were included in this review.

### App description

Basic characteristics of the 36 apps were summarized in Additional file 1: Table S1. Of these 36 unique apps, one was based in Germany, one in South Africa, one in New Zealand, two in Canada, two in Spain, two in Switzerland, three in Scotland, three in the UK, and the remaining 21 in the US.

Fourteen of the 36 apps were paid apps with prices ranging from $0.99 to $6.99. Table 1 shows the number of free and paid apps in each platform, and the price range of paid apps. The median, mean, and standard deviation of paid apps was 2.99, 3.76, and 2.17 for iOS apps. For Android apps, their price was 3.49, 3.38, and 1.44, respectively. There was no significant price difference between iOS apps and Android apps. However, we saw a clear difference between the two platforms in the percentage of free apps. While nearly 74% of Android apps were free, only 48% of iOS apps were free. Note in this table, the removed 5 free lite version apps were added to the 36 unique apps (41 apps in total). The 5 lite version apps and the 5 full version counterparts were counted separately for each platform and the count was shown after the “+” symbol in each table cell. The numbers in front of the “+” symbols are the counts of normal apps that did not have lite versions.

**Table 1:** Counts and price of free and paid apps in each platform

	Android only	iOS only	Cross-platform
Free	10 + 1	5 + 2	7 + 2
Paid	0 + 1	6 + 2	3 + 2
Price	$3.99	$0.99 - $4.99	$0.99 - $6.99

Additional file 1: Table S2 summarizes the pain diary features of the 36 apps into nine categories, which include recordings of pain intensity, pain location, pain quality, pain’s impacts on daily life, other features of pain, other related symptoms, medication, patients’ habits and basic information, and other miscellaneous functions. Among these categories, pain intensity, pain quality, and pain location are essential information about pain. It is noted that 35 out of 36 apps record pain intensity. The only exception was a proprietary app (*Pain Toolkit*) whose information about pain intensity was not available in the description. Twenty-three apps support pain location logging. Sixteen of them include a body map feature and the remaining 7 apps use a fixed list of body parts from which users can select. Only 12 apps support pain quality.

Regarding the pain diary feature, five of the 16 apps could not be evaluated on the test devices (one crashed; two could not register and log in; one was proprietary; one was not available in iOS 11). The remaining 11 functioning apps were used for analysis. Of these 11 apps, five allowed users to directly mark a pain area on the body map by drawing, and five only allowed users to select from predefined body parts. In the remaining app, the body map was only used to display users’ selections from a previous body part list and did not directly allow drawing or selection. These 11 apps used different ways to display pain intensity and pain quality.

The apps are summarized in Table 2, where X indicates an app includes the corresponding feature. As shown in Table 2, 10 apps allowed recording pain intensity on a body map. Only two apps could record pain intensity and pain quality on a body map at the same time. For the five apps that only support selection of body parts, users could not record more than one pain intensity level at the same time. Only one app could record multiple intensity levels and multiple pain qualities at the same time. Of the 11 apps supporting the body map feature, four apps used the pain scale of 0 to 10, four used 1 to 10, two used 1 to 5, and only one used 1 to 6.

**Table 2:** Body map features supported by 11 apps for pain management

App Name	Single Pain Intensity	Multiple Pain Intensity	Single Site Selection	Multiple Site Selection	Single Pain Quality	Multiple Pain Quality
Chronic Pain Tracker	X					
My Pain Diary & Symptom Tracker: Gold Edition	X			X		
My Pain Diary: Chronic Pain & Symptom Tracker	X			X		
My Pain Tracker - Pain Diary		X				
Pain Companion		X				
Pain Diary & Forum CatchMyPain		X				
Pain Tracker & Diary		X				X
PainScale - Pain Diary and Coach	X			X		
Pain Assessment Tool for children	X		X		X	
OurHurt - Chronic Pain						
Pain Tracker HD	X		X			

The impact of pain on daily life is also important information when assessing chronic pain. There were 17 applications supporting this feature. The apps measured the impact in activity level (6), affected activities (3), appetite level (1), bowel movement level (3), fatigue level (5), inability level (1), mood level (11), mobility aid (1), sleep quality (9), social interaction level (1), walking distance (1), and worry about finances (1). The numbers in parenthesis indicate the number of apps with the mentioned feature. Note these numbers are not mutually exclusive since some apps included more than one of these features.

Twenty-four apps collected more features about pain, including pain onset environment (2), factors not triggering pain (1), the change of pain (1), pain description (5), pain duration (6), pain frequency (4), pain can move or not (1), pain onset time (13), pain relieving factors (6), treatments of pain (8), pain triggers (13), and pain type (2).

There were fourteen apps supporting medication recording, including medication adherence (2), duration (1), side effects (2), dosage (9), drug name search (2), drug name browsing (1), drug name free text input (7), OTC drug (3), prescription drug (2), and pain killer usage frequency (2).

Six apps collected patients’ habits and basic health information, including alcohol consumption (1), coffee consumption (2), dairy consumption (1), water consumption (1), diet level (1), time since last meal (1), smoking frequency (1), exercise duration (1), exercise level (1), use of floss (1), sexual activity (1), blood pressure (3), and body weight (3).

Other features of 28 apps were grouped into the miscellaneous category, including audio note (1), note (17), physical therapy (1), find consultant (1), find similar patients (2), important events (2), meaningful activities (1), other comments (3), other factor (1), pain relieving tips (4), pain entry reminders (1), summary charts and plots (19), patient community (2), private messaging (1), and weather (7).

Nineteen apps summarized historical pain diary data by different types of visualizations. Sixteen apps supported line plots with time represented on the x-axis. Thirteen apps used a time interval of day and 3 used an hourly interval. Eight apps had bar charts and three provided pie charts. These counts are not mutually exclusive since some apps use more than one type of visualization.

Additional file 1: Table S3 shows the contents of y-axes in the line plots. It can be seen that pain intensity is supported by 15 apps, followed by mood level (4), pain location (4), other pain-related symptoms (4), medication taking (3), sleep quality (3), weather (3), affected body area (2), fatigue level (2), pain quality (2), activity level (1), body weight (1), events (1), exercise duration (1), pain interference (1), side effects (1).

### Problems in the apps

During the evaluation, two apps crashed repeatedly and three did not allow us to register and log in. These five apps were tested on different devices, but the problem remained. In addition, three iOS apps found from Vionza.com were not available in the App Store of iOS 11. We postulated that they had been discontinued in iOS 11 or earlier versions of iOS. Three apps were proprietary (those apps were publicly available yet do not allow registrations by external users). For these 11 apps, their features were extracted from the text descriptions and screenshots on the app store web pages. Features of the remaining 25 functioning apps were identified.

We found that many apps showed issues with their user interface. For example, some apps did not support browsing or searching drug names. When logging medication, patients had to enter full drug names, which is time-consuming and error-prone. For patients suffering from pain, this can add an extra burden. Some apps contained too many functions on a small screen making the learning curve quite difficult and required users to have enhanced visual acuity and manual dexterity. Since many chronic pain patients have other physical limitations, this must be considered during app development. Other design issues included too small font size, too many colors or patterns on plots.

Regular app updates are imperative for compatibility with their mobile operating system. We found that 21 apps (58%) had not released an update for more than one year. Among them, 14 apps (39%) had not been updated for more than two years. Only 15 apps (42%) had been compiled with the latest application programming interfaces (API).

### HCP and patients involvement

The descriptions and websites of the 36 apps (if available) were checked to determine if healthcare professionals (HCP) and patients were involved in the design and development processes. The results are shown in Additional file 1: Table S4. To differentiate the level of HCP and patient involvement in app development, we created an involvement level. The level is classified into two categories: *systematic* and ad-hoc. The main distinction between them is whether there was direct participation in app development. An example of a *systematic* HCP involvement is embedding pain care experts in app development. It can be seen that HCP were involved in the development of 11 apps (30.6%) and 9 apps out of 11 involved HCP in a systematic way. No app involved patients in app development in a systematic way. Five developers claimed themselves as chronic pain patients (13.9%). Two apps had patients’ input in the design (5.6%). Only one app involved both HCP and patients in the development yet in an ad-hoc way. We found that five apps were created for research purposes or clinical trials (13.9%).

### Apps for clinical use

Last, we checked if any apps fulfilled the four requirements for clinical use. The four requirements are (1) cross-platform compatibility (iOS and Android), (2) clinician access to graphical pain data visualization, (3) HIPAA compliance (or similar one), and (4) PEG survey support. For the first requirement, 12 apps supported both iOS and Android platforms (33.3%). None of the apps supported clinician access to graphical pain data visualization. We could not obtain evidence (based on app descriptions) that pain management apps are HIPAA compliant meaning that no app supported security (or encryption) during data transformation or data storage. None of the apps supported the PEG survey, which was developed for chronic pain assessment and management by primary care physicians.

## Discussion

It is imperative to discuss various features of pain management apps when evaluating them for clinical use. As a result, we will discuss the apps from the viewpoint of app problems, involvement of HCPs and patients in app development, and the clinical usage of pain management apps based on our descriptive evaluation of the pain management apps as well as limitations of the study.

### App problems

There are many issues concerning the apps included in this review. For three apps requiring registration, we could not register because the services were discontinued. Two apps repeatedly crashed on different devices. There are a few possible reasons for these issues. First, developers may have discontinued their services without removing the apps from the two stores. Second, those apps may not have been updated for the latest mobile operating systems (OS). It is imperative for app developers to update their apps for the latest OS. Otherwise, apps do not properly work with the latest OS.

The most serious problem we found is that many apps had not released an update for more than one year, and sometimes more than two years. Less than half of the apps were compatible with the latest mobile operating systems. Additional file 1: Table S5 contains detailed information on these apps. We believe that the 21 apps, especially the 14 apps not updated in two years, should be not used since they have not been maintained and since they might not work properly under the latest or future OS and devices.

### Involvement of HCP and patients in app development

It is imperative for pain-related app developers to involve clinicians in app development to enhance the clinical applicability of app content. Rosser and Eccleston reported in 2011 that the percentage of pain apps with HCP involvement was 14% [16]. Two studies in 2014 and 2015 showed 35 and 8.2% of HCP involvement rates in pain apps, respectively [21, 24]. Our study of pain apps featuring a pain diary function found the percentage was roughly 31% (11/36 apps). This problem has not been improved since Rosser and Eccleston’s study in 2011. A study that reviewed postoperative pain self-management apps in 2017 reported 50% HCP involvement (5/10 apps) [27]. This percentage is much higher than percentages reported in other studies since we believe development of this type of pain app requires specialized medical knowledge.

One interesting finding is that all 11 apps with HCP involvement in development are available in Android. A similar result was reported in 2017 that all postoperative pain self-management apps were available exclusively for Android. Four of these 11 apps (36.4%) are also available in iOS, and there is no iOS-only app developed with HCP involvement.

Although a pain app may include evidence-based and validated clinical content, if the app is not easy-to-use, users have difficulty learning the functions of the app, take more time to accomplish tasks with the app, makes more errors while using it, and thus do not want to use such an app. To improve the ease of use of software, end-users - here patients with chronic pain - should be involved in software development. Only two studies (published in 2014 and 2017) were interested in the involvement of end-users and reported that they could not find any information about end-user involvement in pain apps and in postoperative pain self-management apps, respectively [22, 27]. We found 7 apps (19%) involved end-users in development yet only in an ad-hoc way since we limited our study to pain apps with a pain diary function. Among these seven apps, five (71.4%) of the developers claimed they are chronic pain patients, and the remaining two (28.6%) considered patients’ input. Despite the identification of the seven apps with user involvement, there is no information to what extent HCPs and patients were engaged in the design, making it impossible to describe their influence on the development of these apps.

### Pain management apps for clinical usage

Physicians require access to mobile pain data entered by patients to improve chronic pain management. Ideally, data from a patient’s mobile chronic pain app would be seamlessly uploaded to a secure server connected to the physician’s EMR platform. This way a physician caring for a chronic pain management patient can utilize a graphical interface of a patient’s pain levels across time. The core pain data may be augmented by the remainder of the questions from the validated chronic pain PEG scale, which includes items on interference with function and enjoyment of life. An optimal interface would additionally include data on patient self-administration of pain medication and other relevant events between appointments. These other events may include interventional pain injections, physical therapy, acupuncture, injury, etc. These two sets of data – a patient’s pain levels (or full PEG) and their pain management events – when combined and viewed across time can assist physicians in making data-assisted decisions on the most effective management of a patient’s chronic pain.

To use a pain management app for clinical usage, the app system should support at least two major mobile operating systems (iOS and Android), allow clinicians to access pain data, represent the data in a graphical format and be HIPAA-compliant. We believe these requirements are the minimum for clinical usage. Although many apps support iOS and Android, we could not find any evidence that a single app supports clinician access to graphical pain data visualization and HIPAA compliance. In addition, if a pain management app is used to manage chronic pain by primary care physicians, the app should support the PEG survey tool. PEG support is imperative to primary care physicians because other chronic pain scales such as Chronic Pain Grade questionnaire (CPG) [28] and Brief Pain Inventory (BPI) [29, 30] consist of multiple pages of pain-related questions. It is nearly impractical to adopt the CPG or BPI into a pain management app requesting patients to record daily pain information.

In order to manage chronic pain, physicians must know the pain location, intensity, and quality. We could not find any app that end-users can use to convey these three pieces of pain information in an effective and efficient way. We believe body maps are a great communication tool for chronic pain patients to represent these three pieces of pain information to their physicians by marking (possibly multiple) pain location, intensity, and quality using body maps on a mobile device. With this tool, physicians can quickly obtain detailed chronic pain information using the interface without prolonged conversations with patients.

In this study, we did not perform a formal evaluation of apps for app quality and usability. Such an evaluation can be carried out by adopting a heuristic evaluation providing quick and cost-efficient feedback and insights for usability improvement [35]. A good example is Monkman and Kushniruk’s method for evaluating health app usability [36]. This method contains a set of heuristics consisting of 29 items categorized into 5 groups: Screens (2 items), Content (9), Display (7), Navigation (7), and Interactivity (4) [36]. Our future plan is to utilize such a method for a formal evaluation of pain management apps.

We believe this work contributes to the implementation of mHealth. As a result, based on our findings from this study, we suggest that health-related app developers involve HCP and patients in the development in a systematic way, produce a design aligned with end user characteristics and needs, and regularly update their app (at least once a year). At the same time, end-users should select such apps meeting these requirements.

### Limitations

This review has several limitations. First, in order to search for pain management apps available in Apple App Store, we used a third party service called Vionza.com (rather than Apple App Store), which allows searching, filtering, and categorizing of apps in the App Store. Although the official Apple App Store can be accessed via a web interface from a web browser using a PC, it does not include a search function. The “App Store” app in iOS adopts an extreme recall-focused (or too smart) search mechanism. For example, the searching result for the keyword “pain” contains many “paint” apps and apps in the same category of “paint” apps (even if they do not contain “paint” in their name). In addition, the App Store does not allow for search filtering or sorting. As a result, in order to identify pain management apps for iOS, we used a third-party service. It is unknown how Vionza.com uses Apple App Store data to search for apps. However, based on our experience with Vionza.com, it provides nearly identical search results to the Apple App Store.

Second, several apps could not be tested on devices because they are not open to the public or have quality issues. In this case, their features were extracted from the provided description and screen-captures in the Apple App Store or the Google Play Store. As a result, we might have missed some features in those apps if features were not described or shown in screenshots. Third, this study is specific to English language apps released in the Apple App Store and Google Play Store in the United States. Apps in other languages or stores were not reviewed.

Fourth, when we determined if an app was developed with HCP and/or end-user involvement, we depended entirely on developer websites and app descriptions like the previous 11 app review studies. However, app stores do not verify whether this information is correct. Here, another important issue is raised how their involvement makes a difference in app content and quality. This unresolved issue should be addressed in the near future. Last, since the primary reason for this study was to determine if pain management apps available in the app stores could be used for clinical purposes, we did not perform a quantitative evaluation of the apps or the experience of users. In addition, our app evaluation was basically carried out by a single user.

## Conclusions

Pain management apps including a pain diary function have great potential to enhance pain management outcomes. If a pain management app meets the four requirements for clinical use, and clinicians and patients are involved in app development, those apps will allow augmented monitoring of chronic pain for clinicians and maximum patient participation in their pain management, making pain care more effective and efficient, and therefore improving patient satisfaction with medical care and patient adherence with the treatment plan. In this work, we analyzed 36 pain management apps with the pain diary feature. Many apps demonstrated various problems including security deficiencies. Nearly 60% of the apps had not been updated for more than one year. We believe those apps should not be used for clinical purposes. Most pain apps (around 70%) did not involve clinicians in app development and none of the apps systematically engaged patients with chronic pain as end-users in app development. We could not find any pain apps that are suitable for clinical usage due to lack of HIPAA compliance (or similar one), reasonable clinician support, and inclusion of the validated PEG survey tool for primary care physicians despite high demand from clinicians due to the opioid crisis in the US. The main contribution of this study is our finding that there are no pain management apps designed for clinical use by physicians even though there are many pain management apps available in the app stores. Thus, it is imperative to develop a comprehensive pain management system including an app for patients. That way patients with pain can readily self-report pain data to their provider using an app in a secure manner, and clinicians can monitor patients’ pain for immediate medical intervention and/or adjustment of pain medication. Future research should develop uniform reporting procedures consisting of a set of software and medical requirements as a potential methodology for pain management app evaluation.

## Additional file

Additional file 1: **Table S1.** Basic information of 36 mobile applications for pain management. **Table S2.** Pain diary features of 36 mobile applications for pain management. **Table S3.** Contents of line plots supported by 16 mobile applications for pain management. **Table S4.** HCP and patients’ involvement in design. **Table S5.** Last version update dates (as of May 2018). (DOCX 25 kb)

## Data Availability

Not applicable.

## Abbreviations

API: Application programming interfaces
BPI: Brief Pain Inventory
CDC: US Centers for Disease Control and Prevention
CPG: Chronic Pain Grade questionnaire
EMR: Electronic medical record
HCP: Healthcare Professionals
HIPAA: Health Insurance Portability and Accountability Act
OTC: Over-the-counter
PEG: Pain Intensity, Enjoyment of life, General activity
