# Supplementary material for: Mobile applications for pain management: an app analysis for clinical usage
Source: BMC Med Inform Decis Mak. 2019 May 30;19:106. doi: 10.1186/s12911-019-0827-7 (PMC6543581; doi:10.1186/s12911-019-0827-7)
Supplement: Supplementary file 1 — Table S1. Basic information of 36 mobile applications for pain management. Table S2. Pain diary features of 36 mobile applications for pain management. Table S3. Contents of line plots supported by 16 mobile applications for pain management. Table S4. HCP and patients’ involvement in design. Table S5. Last version update dates (as of May 2018). (DOCX 25 kb) [file 12911_2019_827_MOESM1_ESM.docx]

| **Application Name in iOS** | **Application Name in Android** | **Developer** | **Country** | **Price**  **(iOS)** | **Price (Android)** |
| --- | --- | --- | --- | --- | --- |
| ACPA Pain Logs | ACPA Pain Logs | American Chronic Pain Association | USA | Free | Free |
| Chronic Pain Diary |  | Ben Delaporte | USA | Free | N/A |
|  | Chronic Pain Diary | Jet5 | Scotland | N/A | Free |
| Chronic Pain Tracker |  | Chronic Stimulation, LLC | USA | US$6.99 | N/A |
|  | Chronicle: Chronic Symptom and Pain Tracker | UpandAtom Applications | USA | N/A | Free |
| EPM - Essential Pain Management | Essential Pain Management | HealthObs, Ltd | New Zealand | Free | Free |
| FibroMapp | FibroMapp Pain Manager+ | Bodymap Apps | Scotland | US$2.99 | US$2.84 |
| Keele Pain Recorder | Keele Pain Recorder | Keele University | UK | Free | Free |
|  | Manage My Pain | ManagingLife | Canada | N/A | US$3.99 |
| My Pain Diary & Symptom Tracker: Gold Edition |  | Damon Lynn | USA | US$4.99 | N/A |
| My Pain Diary: Chronic Pain & Symptom Tracker | My Pain Diary | Damon Lynn | USA | US$4.99 | US$4.99 |
| My Pain Logs |  | Subinprara infotech, Inc | USA | Free | N/A |
| My Pain Tracker - Pain Diary |  | Merc App Solutions, LLC | USA | Free | N/A |
| Ouch - Chronic Pain Log, Migraine Diary |  | GammaPoint, LLC | USA | US$1.99 | N/A |
|  | OurHurt - Chronic Pain | Labs Health Company | USA | N/A | Free |
|  | Pain Assessment Tool for children | Bcom | South Africa | N/A | Free |
|  | Pain Buddy | MSERV | UK | N/A | Free |
| Pain Care | Pain Care | Ringful, LLC | USA | Free | Free |
|  | Pain Clinic | Aventura | USA | N/A | Free |
| Pain Companion | Pain Companion | Sanovation AG | Switzerland | Free | Free |
| Pain Diary & Forum CatchMyPain | Pain Diary & Forum CatchMyPain | Sanovation AG | Switzerland | US$6.99 | US$4.49 |
|  | Pain Log | Raúl R. | Spain | N/A | Free |
| Pain Logger |  | Talon Strike Software | USA | US$1.99 | N/A |
| Pain Management |  | Prime Technology Group, Inc | USA | Free | N/A |
|  | Pain Monitor 2.0 | Azucena Garcia-Palacios | Spain | N/A | Free |
| Pain Scale - your digital log for chronic pain |  | odeesoft | Germany | US$5.99 | N/A |
| Pain Toolkit | Pain Toolkit | Advanced Digital Institute - Health | UK | Free | Free |
| Pain Tracker |  | iHealth Ventures, LLC | USA | US$0.99 | N/A |
| Pain Tracker - Daily Symptom Diary |  | Pixel Mafia, LLC | USA | US$1.99 | N/A |
| Pain Tracker & Diary | Pain Tracker & Diary | Nanolume, LLC | USA | US$2.99 | US$2.99 |
|  | Pain Tracker HD | AppYourWay | Canada | N/A | Free |
| PainScale - Pain Diary and Coach | PainScale - Chronic Pain Coach | Boston Scientific | USA | Free | Free |
| PainTrackr - Track Pain, Chronic Pain, and Pain History |  | Involution Studios | USA | Free | N/A |
| Tame My Pain | Tame My Pain | Raj Navani | USA | US$0.99 | US$0.99 |
|  | The Pain App | Social Care Alba | Scotland | N/A | Free |
| Symptom Tracker by TracknShare |  | Track & Share Apps, LLC | USA | US$4.99 | N/A |

Table S1. Basic information of 36 mobile applications for pain management.

Note: Cross-platform applications have both iOS and Android names. If an application has only one name, it is only available in that platform.

Table S2. Pain diary features of 36 mobile applications for pain management.

| **Application Name** | **Pain intensity** | **Pain location** | **Pain quality** | **Impacts on life** | **Other pain features** | **Other symptoms** | **Medication taking** | **Patient health information** | **Miscellaneous** |
| --- | --- | --- | --- | --- | --- | --- | --- | --- | --- |
| ACPA Pain Logs | X |  |  | X | X | X |  | X |  |
| Chronic Pain Diary (by Ben Delaporte) | X |  |  |  | X | X |  |  | X |
| Chronic Pain Diary (by Jet5) | X | X | X | X | X | X |  |  | X |
| Chronic Pain Tracker | X | X |  | X | X | X | X | X | X |
| Chronicle: Chronic Symptom and Pain Tracker | X |  | X |  | X | X | X |  | X |
| EPM - Essential Pain Management | X |  |  | X | X |  |  |  | X |
| FibroMapp | X | X | X | X | X | X | X |  | X |
| Keele Pain Recorder | X |  | X | X |  |  |  |  | X |
| Manage My Pain | X | X | X |  | X | X |  |  | X |
| My Pain Diary & Symptom Tracker: Gold Edition | X | X | X | X | X | X | X | X | X |
| My Pain Diary: Chronic Pain & Symptom Tracker | X | X | X | X | X |  | X | X | X |
| My Pain Logs | X | X | X | X | X | X | X |  | X |
| My Pain Tracker - Pain Diary | X | X |  |  | X | X | X |  | X |
| Ouch - Chronic Pain Log, Migraine Diary | X | X |  |  | X | X | X |  | X |
| OurHurt - Chronic Pain | X | X |  | X | X |  |  |  | X |
| Pain Assessment Tool for children | X | X | X |  | X |  |  |  |  |
| Pain Buddy | X |  |  |  |  |  |  |  | X |
| Pain Care | X |  |  |  | X |  | X |  | X |
| Pain Clinic | X |  |  | X |  |  |  |  |  |
| Pain Companion | X | X |  | X | X | X |  |  | X |
| Pain Diary & Forum CatchMyPain | X | X | X |  | X |  | X |  | X |
| Pain Log | X |  |  |  | X |  |  |  | X |
| Pain Logger | X | X |  |  |  |  |  |  | X |
| Pain Management | X | X |  |  |  |  |  |  | X |
| Pain Monitor 2.0 | X |  |  | X |  |  |  |  |  |
| Pain Scale - your digital log for chronic pain | X |  |  |  | X |  |  |  | X |
| Pain Toolkit |  | X |  |  |  |  |  |  |  |
| Pain Tracker | X | X |  |  | X |  |  |  | X |
| Pain Tracker - Daily Symptom Diary | X |  |  |  |  |  |  |  |  |
| Pain Tracker & Diary | X | X | X |  |  |  |  |  | X |
| Pain Tracker HD | X | X |  | X | X |  | X | X | X |
| PainScale - Pain Diary and Coach | X | X |  | X | X | X | X |  | X |
| PainTrackr - Track Pain, Chronic Pain, and Pain History | X | X |  |  |  |  |  |  |  |
| Tame My Pain | X | X | X | X | X |  |  |  |  |
| The Pain App | X | X |  |  |  |  | X |  | X |
| Symptom Tracker by TracknShare | X |  |  | X |  | X | X | X | X |

Table S3. Contents of line plots supported by 16 mobile applications for pain management.

| **Application Name** | **Activity level** | **Affected body area** | **Body weight** | **Events** | **Exercise duration** | **Fatigue level** | **Medication taking** | **Mood level** | **Other symptoms** | **Pain intensity** | **Pain interference** | **Pain location** | **Pain quality** | **Side effect** | **Sleep quality** | **Weather** |
| --- | --- | --- | --- | --- | --- | --- | --- | --- | --- | --- | --- | --- | --- | --- | --- | --- |
| Chronic Pain Diary (by Ben Delaporte) |  |  |  |  |  |  |  |  |  | X |  |  |  |  |  |  |
| Chronic Pain Diary (by Jet5) |  |  |  |  |  |  |  |  |  | X |  | X |  |  |  |  |
| Chronic Pain Tracker | X |  |  |  |  |  |  |  |  | X |  |  |  |  |  |  |
| Chronicle: Chronic Symptom and Pain Tracker |  |  |  | X |  |  | X |  | X | X |  |  |  |  |  |  |
| FibroMapp |  |  |  |  |  |  |  |  |  |  |  |  |  |  | X |  |
| Keele Pain Recorder |  |  |  |  |  |  | X | X |  | X | X |  |  | X | X |  |
| Manage My Pain |  |  |  |  |  |  |  |  |  | X |  |  |  |  |  |  |
| My Pain Diary & Symptom Tracker: Gold Edition |  |  |  |  |  |  |  |  |  | X |  | X | X |  |  | X |
| My Pain Diary: Chronic Pain & Symptom Tracker |  |  |  |  |  |  |  |  |  | X |  | X | X |  |  | X |
| My Pain Tracker - Pain Diary |  |  |  |  |  |  |  |  |  | X |  | X |  |  |  |  |
| Pain Companion |  |  |  |  |  | X |  | X | X | X |  |  |  |  |  |  |
| Pain Diary & Forum CatchMyPain |  | X |  |  |  | X |  | X | X | X |  |  |  |  |  |  |
| Pain Logger |  |  |  |  |  |  |  |  |  | X |  |  |  |  |  |  |
| Pain Tracker & Diary |  | X |  |  |  |  |  |  |  | X |  |  |  |  |  |  |
| PainScale - Pain Diary and Coach |  |  |  |  |  |  |  |  |  | X |  |  |  |  |  |  |
| Symptom Tracker by TracknShare |  |  | X |  | X |  | X | X | X | X |  |  |  |  | X | X |

| **Application Name** | **HCP Involvement in Design** | **Patient Involvement in Design** | **For Research or Clinical Trials** |
| --- | --- | --- | --- |
|  |  |  |  |
| ACPA Pain Logs | X (systematic) |  |  |
| Chronic Pain Diary (by Ben Delaporte) |  |  |  |
| Chronic Pain Diary (by Jet5) | X (systematic) |  |  |
| Chronic Pain Tracker |  | X (ad-hoc) |  |
| Chronicle: Chronic Symptom and Pain Tracker |  |  |  |
| EPM - Essential Pain Management | X (systematic) |  |  |
| FibroMapp |  | X (ad-hoc) |  |
| Keele Pain Recorder | X (systematic) |  | X |
| Manage My Pain | X (ad-hoc) |  |  |
| My Pain Diary & Symptom Tracker: Gold Edition |  | X (ad-hoc) |  |
| My Pain Diary: Chronic Pain & Symptom Tracker |  | X (ad-hoc) |  |
| My Pain Logs |  |  |  |
| My Pain Tracker - Pain Diary |  |  |  |
| Ouch - Chronic Pain Log, Migraine Diary |  |  |  |
| OurHurt - Chronic Pain | X (systematic) |  |  |
| Pain Assessment Tool for children |  |  |  |
| Pain Buddy | X (systematic) |  | X |
| Pain Care | X (systematic) |  |  |
| Pain Clinic |  |  | X |
| Pain Companion |  | X (ad-hoc) |  |
| Pain Diary & Forum CatchMyPain | X (systematic) |  | X |
| Pain Log |  |  |  |
| Pain Logger |  |  |  |
| Pain Management |  |  |  |
| Pain Monitor 2.0 | X (systematic) |  | X |
| Pain Scale - your digital log for chronic pain |  |  |  |
| Pain Toolkit |  | X (ad-hoc) |  |
| Pain Tracker |  |  |  |
| Pain Tracker - Daily Symptom Diary |  |  |  |
| Pain Tracker & Diary |  |  |  |
| Pain Tracker HD |  |  |  |
| PainScale - Pain Diary and Coach | X (ad-hoc) | X (ad-hoc) |  |
| PainTrackr - Track Pain, Chronic Pain, and Pain History |  |  |  |
| Tame My Pain |  |  |  |
| The Pain App |  |  |  |
| Symptom Tracker by TracknShare |  |  |  |

Table S4. HCP and patients’ involvement in design.

| **Application Name** | **Latest Version/Update Date** | | | |
| --- | --- | --- | --- | --- |
|  | **Android** | | **iOS** | |
| ACPA Pain Logs | 1.1.14 | 2/23/2017 | 1.1.0 | 3/13/2017 |
| Chronic Pain Diary (by Ben Delaporte) |  |  | 1.21 | 1/17/2017 |
| Chronic Pain Diary (by Jet5) | 1.01 | 1/31/2014 |  |  |
| Chronic Pain Tracker |  |  | 3.8.8 | 6/5/2017 |
| Chronicle: Chronic Symptom and Pain Tracker | 1.50 | 4/3/2018 |  |  |
| EPM - Essential Pain Management | 1.0 | 2/22/2017 | 2.0.1 | 11/21/2016 |
| FibroMapp | 1.2.3 | 11/5/2013 | 1.6 | 11/13/2016 |
| Keele Pain Recorder | 1.034 | 2/14/2018 | 1.02 | 2/14/2018 |
| Manage My Pain | varies w/ device | 3/5/2018 |  |  |
| My Pain Diary & Symptom Tracker: Gold Edition |  |  | 1.1.0 | 3/19/2017 |
| My Pain Diary: Chronic Pain & Symptom Tracker | 1.9.2 | 5/15/2016 | 3.5.8 | 3/10/2016 |
| My Pain Logs |  |  | 1.8.1 | 4/1/2016 |
| My Pain Tracker - Pain Diary |  |  | 4.7 | 11/20/2017 |
| Ouch - Chronic Pain Log, Migraine Diary |  |  | 1.0 | 12/17/2013 |
| OurHurt - Chronic Pain | 1.2 | 2/25/2014 |  |  |
| Pain Assessment Tool for children | 1.1.1 | 9/15/2017 |  |  |
| Pain Buddy | 1.1 | 11/9/2017 |  |  |
| Pain Care | 1.4 | 10/29/2010 | 2.4 | 10/29/2010 |
| Pain Clinic | 0.16.00-Pain | 4/28/2018 |  |  |
| Pain Companion | 4.1.0 | 9/30/2016 | 4.1.0 | 9/30/2016 |
| Pain Diary & Forum CatchMyPain | 3.5.6 | 3/8/2016 | 3.5.5 | 3/10/2016 |
| Pain Log | 1.2.1 | 3/29/2015 |  |  |
| Pain Logger |  |  | 2.4 | 11/30/2016 |
| Pain Management |  |  | 1.0 | 5/13/2014 |
| Pain Monitor 2.0 | 1.0 | 9/18/2017 |  |  |
| Pain Scale - your digital log for chronic pain |  |  | 2.1 | 4/26/2018 |
| Pain Toolkit | 2.0.19.1260 | 4/7/2017 | 2.9 | 4/10/2017 |
| Pain Tracker |  |  | 2.5 | 1/4/2018 |
| Pain Tracker - Daily Symptom Diary |  |  | 1.0 | 1/26/2016 |
| Pain Tracker & Diary | 1.51 | 4/24/2018 | 3.8 | 12/14/2017 |
| Pain Tracker HD | 1.1.0 | 1/28/2017 |  |  |
| PainScale - Pain Diary and Coach | 2.1.1 | 5/2/2018 | 2.1 | 4/26/2018 |
| PainTrackr - Track Pain, Chronic Pain, and Pain History |  |  | 1.1 | 4/21/2013 |
| Tame My Pain | 1.0.6 | 4/8/2016 | 1.0.6 | 4/12/2016 |
| The Pain App | 0.9 | 9/30/2012 |  |  |
| Symptom Tracker by TracknShare |  |  | 7.4.2 | 11/29/2017 |

Table S5. Last version update dates (as of May 2018)
